# An equation for estimating low-density lipoprotein-triglyceride content and its use for cardiovascular disease risk stratification

**DOI:** 10.3389/fcvm.2024.1452869

**Published:** 2024-10-25

**Authors:** Anna Wolska, Maureen Sampson, Rafael Zubirán, Jeff W. Meeusen, Leslie J. Donato, Allan S. Jaffe, Alan T. Remaley

**Affiliations:** ^1^ Lipoprotein Metabolism Laboratory, Translational Vascular Medicine Branch, National Heart, Lung, and Blood Institute, National Institutes of Health, Bethesda, MD, United States; ^2^ Department of Laboratory Medicine, Clinical Center, National Institutes of Health, Bethesda, MD, United States; ^3^ Department of Laboratory Medicine and Pathology, Mayo Clinic, Rochester, MN, United States

**Keywords:** ASCVD, cardiovascular disease, LDL-cholesterol, LDL-triglycerides, risk marker, risk score, triglycerides

## Abstract

**Background:**

The triglyceride (TG) content of low-density lipoprotein (LDL-TG) has been shown to be more predictive of atherosclerotic cardiovascular disease (ASCVD) events than the cholesterol content of LDL (LDL-C). The goal of our study was to develop an equation for estimating LDL-TG (*e*LDL-TG) based on the standard lipid panel and to compare it to estimated LDL-C as an ASCVD risk biomarker.

**Methods:**

Using least-square regression analysis, the following *e*LDL-TG equation was developed: $e\mathrm{LDL}\text{-}\mathrm{TG}=\frac{\mathrm{TG}}{38.5}+\frac{\mathrm{NonHDL}\text{-}C}{5.75}+\frac{9.75\;\mathrm{TG}}{\mathrm{NonHDL}\text{-}C}+\frac{244}{\mathrm{HDL}\text{-}C}-2.95$. LDL-TG was measured by the *β*-quantification (BQ) reference method (*N* = 40,202). LDL-C was calculated by the Sampson-NIH equation. The association of LDL-C and *e*LDL-TG with ASCVD risk markers was performed in the National Heart and Nutrition Examination Survey (NHANES) (*N* = 37,053) and with ASCVD events in a primary prevention cohort from the UK Biobank (UKB) (*N* = 429,367) and the Atherosclerosis Risk in Communities (ARIC) study (*N* = 14,632).

**Results:**

*e*LDL-TG showed better ASCVD risk stratification of UKB participants than LDL-C (Wilcoxon Chi-Square: 2,099.6 vs. 418.7, respectively). Receiving-operating characteristics analysis revealed that *e*LDL-TG had a stronger association with ASCVD events than LDL-C (AUC: 0.596 vs. 0.542, respectively) and other conventional lipid markers. Similar findings were found in ARIC. Discordance analysis in UKB showed that the group with low LDL-C/high *e*LDL-TG had a similar risk as the high LDL-C/high *e*LDL-TG group. Furthermore, these same two groups with the highest *e*LDL-TG levels and the highest ASCVD event rate also had higher mean levels of systolic blood pressure, Body Mass Index, hemoglobin A1C, and C-reactive protein than the two lower *e*LDL-TG groups. Using *e*LDL-TG > 44.6 mg/dl (80th percentile) as a cut-point leads to a hazard ratio of 1.32 (95% CI, 1.29–1.36) for ASCVD events, which remained significant after adjustment for LDL-C and apoB. Furthemore, using *e*LDL-TG as a risk-enhancer test leads to reclassification of 50% more high-risk individuals than current lipid-enhancer test rules.

**Conclusions:**

Like LDL-C, LDL-TG can also be calculated from the results of the standard lipid panel. Compared to estimated LDL-C, *e*LDL-TG was a better risk marker for primary prevention and hence could improve initial ASCVD risk stratification.

## 1 Introduction

Increased cholesterol on low-density lipoproteins (LDL-C) is a key causal factor in the development of atherosclerotic cardiovascular disease (ASCVD) (1). Hence, LDL-C is the primary therapeutic target for ASCVD risk reduction, a strategy long endorsed in the US (2) and elsewhere (3). A significant residual ASCVD risk persists, however, after statin treatment, particularly for those patients with elevated triglyceride (TG) concentrations (4).

LDL particles transport not only cholesterol but also other lipids, such as triglycerides (5, 6). Although other larger apoB-containing lipoproteins like chylomicrons and very low-density lipoproteins (VLDL) are the main carriers of TG, substantial amounts of TG can remain on LDL from incomplete lipolysis or can be transferred to LDL from TG-rich lipoproteins (TRL) via cholesteryl-ester transfer protein (CETP) in an exchange of cholesteryl esters (6, 7). Typically, about 6% of total plasma TG are found on LDL (5) but with some types of dyslipidemias a considerable greater fraction of TG can be present on LDL. Impaired lipolysis and delayed clearance of TRL, which occurs in several high-risk ASCVD conditions associated with hypertriglyceridemia, such as obesity, metabolic syndrome, and diabetes, can lead to the enrichment of TG on LDL (5, 8). TG-enriched LDL particles can undergo further lipolysis, leading to the generation of small dense LDL (*sd*LDL), which is associated with increased ASCVD risk (6, 9). Recently, a direct assay for cholesterol on *sd*LDL has been developed (10) and in multiple studies it was more strongly predictive of future ASCVD events than total LDL-C (11–14).

One of the first studies to report that the TG content of LDL (LDL-TG) may be a useful predictor of ASCVD risk was by Marz et al. in 2004 (15). The results of their cross-sectional study of patients with stable coronary artery disease (CAD) from Ludwigshafen Risk and Cardiovascular Health study showed that LDL-TG, as measured by the *β*-quantification (BQ) reference method, was more strongly associated with prevalent CAD than LDL-C. LDL-TG was also found to have a greater association with systemic low-grade inflammation than LDL-C. LDL-TG measured by a new direct assay in the Atherosclerosis Risk in Communities (ARIC) study was reported in 2018 to be an independent predictor of ASCVD events and also superior to LDL-C (16). In another recent study, two large prospective cohorts from a large European population study also found that LDL-TG measured by a direct assay was more strongly associated with increased ASCVD events than LDL-C, which was further supported by meta-analyses of several previous studies (17). Bayesian network analysis of patients examined for atherosclerosis by cardiac computed tomography indicated that LDL-TG could possibly be causally related to the development of cardiovascular disease (18).

There are several possible methods for measuring LDL-TG, although none are commonly performed in routine clinical practice. The first is by the BQ reference method, which is based on a combination of density gradient ultracentrifugation and LDL precipitation. If both cholesterol and TG is measured in each fraction generated by this method, one can measure not only LDL-C but also LDL-TG (15). Most of the recent studies on LDL-TG (16–18) have used an automated direct assay produced by Denka (19), but this assay is not yet approved by the FDA. There is also a lipoprotein fractionation method offered by a commercial reference laboratory based on high-performance liquid chromatography, which measures both cholesterol and TG in all separated lipoprotein fractions, including LDL (20).

Given the recent promising data on the potential value of LDL-TG as an ASCVD biomarker but the current limited options for its measurement, we investigated whether one could possibly estimate LDL-TG from the results of the standard lipid panel similar to what is routinely done for LDL-C (5). We describe here a simple equation for estimating LDL-TG (*e*LDL-TG) that matches LDL-TG by the BQ reference method (*BQ*LDL-TG). Furthermore, we show that *e*LDL-TG is more strongly associated with ASCVD risk than estimated LDL-C or directly measured LDL-C in several primary prevention cohorts.

## 2 Materials and methods

Deidentified lipid and apolipoprotein B (apoB) test results were obtained from the Mayo Clinic on patients (*N* = 40,349) for whom BQ testing was performed for routine clinical care. Lipid testing for BQ were performed on a Roche cobas instrument (cholesterol-enzymatic method, triglycerides-enzymatic method, HDL-C-dextran sulfate precipitation method). ApoB levels were measured in a subset of this population (*N* = 24,406) using an immunoturbidometric assay performed on a Cobas c501 analyzer (Roche Diagnostics, IN). Samples with detectable Lipoprotein-X based on agarose gel electrophoresis (*N* = 147) were excluded from analysis. Deidentified lipid test results from Nuclear Magnetic Resonance (NMR) spectroscopy analysis (21) (*N* = 13,788) were obtained from the NIH to compare *e*LDL-TG concentrations with LDL particle number and size. Fasting lipid panel test results (*N* = 37,053) from years 2005 to 2020 were downloaded from the National Health and Nutrition Examination survey (NHANES) (https://wwwn.cdc.gov/nchs/nhanes/) and used to evaluate *e*LDL-TG as a risk-enhancer test. No exclusion criteria based on age, drug use, or lipid values were applied to this cohort unless when indicated. To investigate the association of *e*LDL-TG with ASCVD events, test results and demographic information were downloaded for participants with a negative history of ASCVD from the Atherosclerosis Risk in Communities (ARIC) study (*N* = 14,632) (https://biolincc.nhlbi.nih.gov/studies/aric/) and from the UK Biobank (UKB) (*N* = 429,367). Individuals who were on lipid-lowering medication on the first visit (ARIC: *N* = 437, UKB: *N* = 75,023) or with incomplete follow-up data (UKB: *N* = 82,584) were excluded from analysis. In UKB cohort, ASCVD events over a 14-year period were defined either by International Classification of Diseases (ICD) codes (ICD-10: I20, I21, I22, I23, I24, I25, I63, I64, I65, I66, I70) or by the corresponding algorithmically defined myocardial infarction (MI) and stroke outcomes. For ARIC, only baseline lipid results from the first study visit were used for analysis and ASCVD was defined as including one of the following events: fatal and non-fatal MI and stroke. A summary of the lipid values and demographic information of the different populations used in this study are shown in Supplementary Table 1.

An equation for *e*LDL-TG based on the BQ reference method was developed by least-square regression analysis. The maximum lipid values used to develop the equation are the following: HDL-C = 201 mg/dl, TC = 1,830 mg/dl, TG = 11,950 mg/dl, *BQ*LDL-C = 1,775 mg/dl, *BQ*LDL-TG = 1,016 mg/dl. LDL-C concentrations were calculated by the Sampson/NIH equation (22). *sd*LDL-C was calculated as previously described (14) and remnant cholesterol (Rem-C) was calculated by subtracting LDL-C from nonHDL-C. The 10-year ASCVD risk was calculated as previously described (23) using the pooled cohort equations (PCE) risk score. BQ data from the Mayo Medical Laboratories (MML) dataset were randomly split into a training (*N* = 20,191) and validation (*N* = 20,011) datasets. The training dataset was used to initially develop the *e*LDL-TG equation, which was then tested on the validation dataset.

Kaplan-Meier survival curves with Wilcoxon Chi-Square analysis were performed to compare time to ASCVD events for patients by quintiles of various lipid parameters. Logistic regression was performed against ASCVD events to obtain receiver-operating characteristic (ROC) curves, which were quantified by the area under the curve (AUC). The association of each lipid variable with incident ASCVD was tested using Cox proportional hazard models. Models for the incident ASCVD event were adjusted for nonlipid variables comparable to those used in the American College of Cardiology/American Heart Association guidelines: age, sex, race, systolic blood pressure (SBP), blood pressure medication (BP meds), diabetes, and smoking. All statistical analyses were done using JMP software (JMP, Cary, NC) or by Excel (Microsoft, Redmond, WA). Software for estimating *e*LDL-TG by the newly developed equation can be freely downloaded at the Figshare website (website: https://doi.org/10.6084/m9.figshare.23679375). Research under this study was not considered human subject research and was exempted from IRB review.

## 3 Results

### 3.1 Development of a new equation for calculating LDL-TG

**Figure 1:**
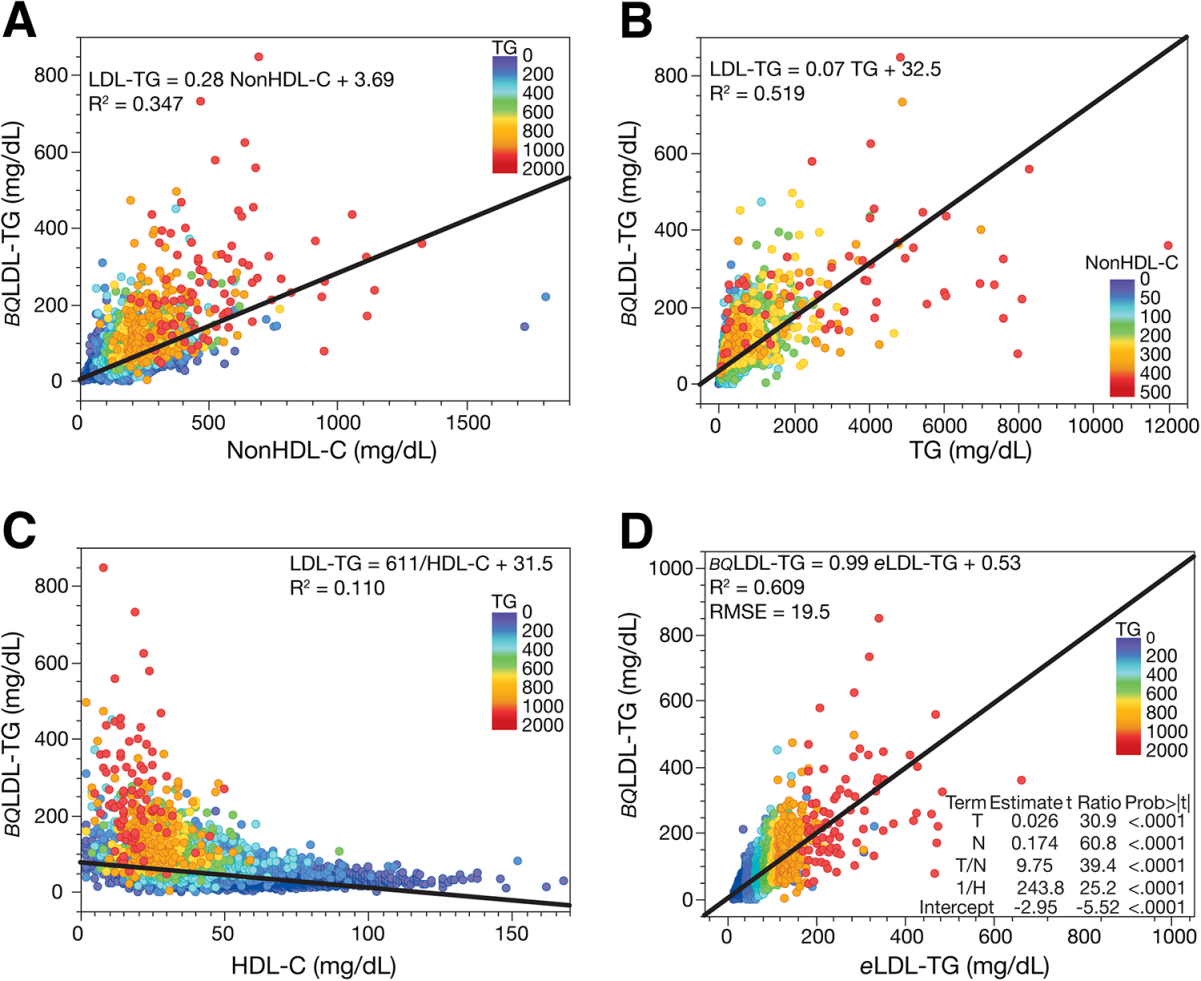
Correlation of *BQ*LDL-TG with lipid parameters and validation of *e*LDL-TG equation. Relationship between LDL-TG measured by the BQ reference method (*BQ*LDL-TG) (*N* = 40,202) and nonHDL-C **(Panel A)**, TG **(Panel B)**, and HDL-C **(Panel C)**. Development of the equation for estimated LDL-TG (*e*LDL-TG) **(Panel D)**. Equation parameters are from the training dataset (*N* = 20,191) and graphed results are from the validation dataset (*N* = 20,011). Results are colored by TG **(Panels A, C, and D)** and nonHDL-C **(B)**.

Using a large general population cohort (*N* = 40,202), we examined the relationship between the different test results from the standard lipid panel with LDL-TG concentrations, as measured by BQ reference method (*BQ*LDL-TG) (Figure 1). Both non-high-density lipoprotein-cholesterol (nonHDL-C) (Figure 1A) and TG (Figure 1B) were positively correlated with *BQ*LDL-TG, but there was a closer correlation to TG. In contrast, high-density-lipoprotein-cholesterol (HDL-C) was inversely related to *BQ*LDL-TG (Figure 1C). Apolipoprotein B (apoB) also had a weak positive association with LDL-TG (Supplementary Figure 1), but because it is currently not often used in routine clinical care and not part of the standard lipid panel, it was not further utilized.

Based on the relationships we observed in Figure 1A–C, we developed by least-squares regression analysis the following equation for estimating LDL-TG concentrations:

$$e\mathrm{LDL}\text{-}\mathrm{TG}=\frac{\mathrm{TG}}{38.5}+\frac{\mathrm{NonHDL}\text{-}C}{5.75}+\frac{9.75\;\mathrm{TG}}{\mathrm{NonHDL}\text{-}C}+\frac{244}{\mathrm{HDL}\text{-}C}-2.95$$

Although the final equation, which utilized 3 different lipid variables, showed a closer relationship to LDL-TG than any of the individual lipid variables, the overall correlation of *e*LDL-TG with *BQ*LDL-TG was still only relatively modest (*R*^2^ = 0.609) (Figure 1D). Nearly identical results were obtained for both the training and validation dataset, indicating that the equation was not overfitted. The *e*LDL-TG equation did yield a slope close to 1.0 and an intercept of nearly zero when compared to *BQ*LDL-TG. Inclusion of higher order mathematical terms did not substantially improve the fit. Based on the magnitude of the error observed by residual error plots (Supplementary Figure 2), the *e*LDL-TG equation should be restricted to samples with TG < 1,000 mg/dl (<11.3 mmol/L) and nonHDL-C < 400 mg/dl (<10.36 mmol/L). A version of the *e*LDL-TG equation in SI units can be found in the supplement (Supplementary Figure 3).

### 3.2 Evaluation of the association of *e*LDL-TG with ASCVD events

**Figure 2:**
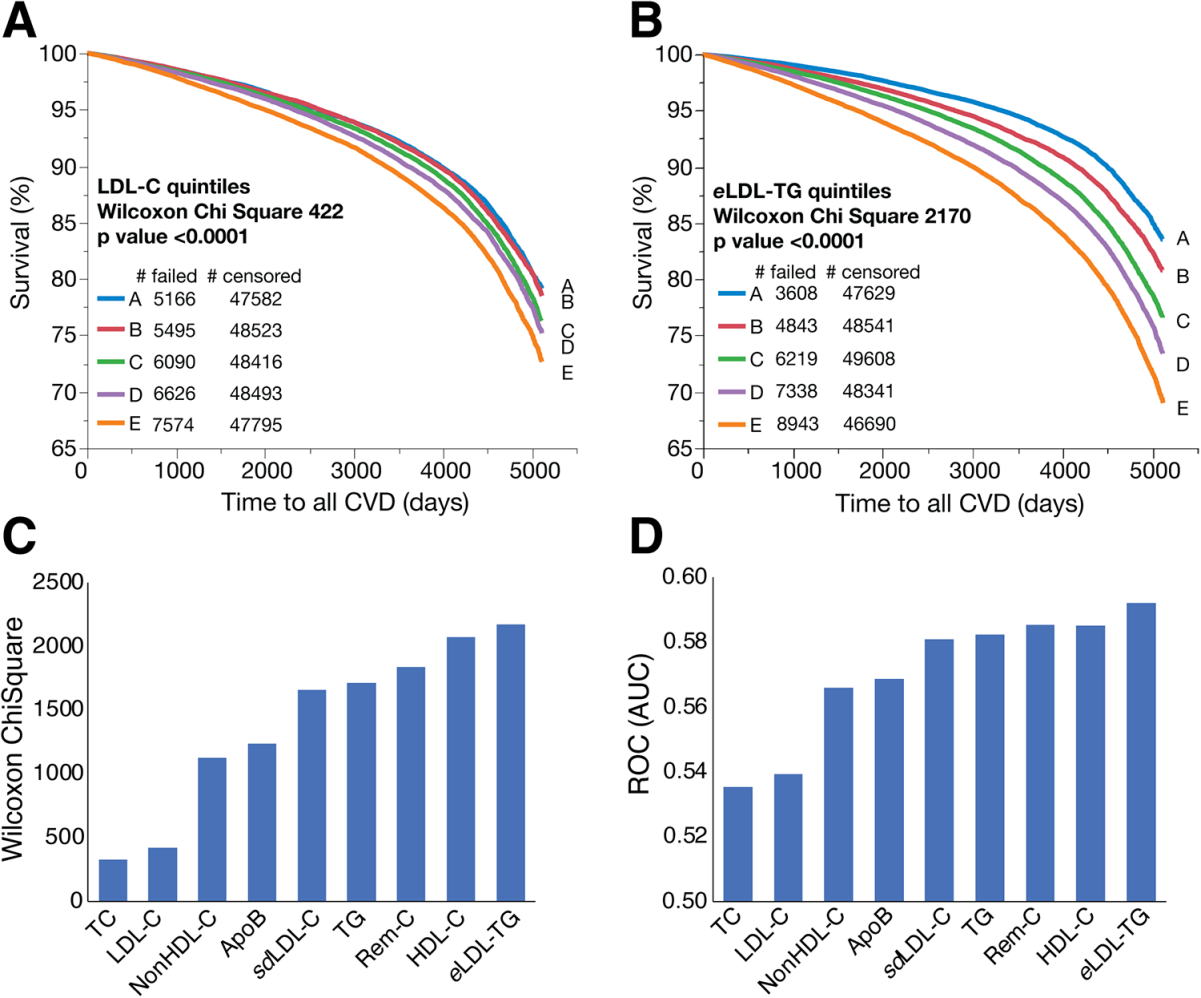
Kaplan-Meier survival curves and lipid test evaluation for ASCVD events in the UKB dataset. Kaplan-Meier survival curves in the UKB dataset for people without any lipid medications or incomplete follow-up data (*N* = 271,760) for all ASCVD events were calculated for LDL-C quintiles **(Panel A)** and *e*LDL-TG quintiles **(Panel B)**. Other lipid tests were divided into quintiles for survival curves and the Wilcoxon Chi-Square is ranked **(Panel C)**. The same set of lipid tests was evaluated with logistic regression for ASCVD and the AUC is ranked **(Panel D)**.

Using a cohort of primary prevention participants in UKB not on any lipid-lowering medications (*N* = 271,760), we performed survival curve and Cox proportional hazards analysis for all ASCVD events. Compared to LDL-C quintiles (Figure 2A), grouping patients into *e*LDL-TG quintiles (Figure 2B) showed much better risk stratification and resulted in greater separation between low and high risk subjects. Based on its overall Chi-square score (Figure 2C), *e*LDL-TG was superior to LDL-C and other commonly used biomarkers of pro-atherogenic lipoproteins and also slightly better than HDL-C as a predictor of future ASCVD events. Notably, *e*LDL-TG was more strongly associated with ASCVD events than Rem-C, *sd*LDL-C and apoB. Similar findings were found after ROC analysis for predicting future ASCVD events (Figure 2D). Using a cutoff for *e*LDL-TG of 44.6 mg/dl (80th perecentile), the risk for future ASCVD was increased with an adjusted hazards ratio (HR) of 1.32 (95% CI, 1.29–1.36), and it remained significant after normalizing for LDL-C or apoB (HR of 1.25 [95% CI, 1.22–1.28] and 1.14 [95% CI, 1.11–1.18], respectively).

When compared LDL-C, *e*LDL-TG continued to be a superior marker in both unadjusted and adjusted analysis. The unadjusted HR per 1-SD change of *e*LDL-TG was greater than LDL-C (HR per 1-SD of 1.24 [95% CI, 1.23–1.25] and 1.11 [95% CI, 1.10–1.12], respectively). This stronger association remained significant even after adjustment for age, sex, SBP, BP meds, ethnicity, smoking, diabetes and body mass index (BMI) with a HR of 1.15 [95% CI, 1.14–1.16] and 1.12 [95% CI, 1.11–1.13], respectively. We performed a similar analyses in the ARIC cohort (*N* = 14,195) and confirmed that *e*LDL-TG is a better risk marker of ASCVD than LDL-C or other conventional lipid tests (Supplementary Figure 4). Interestingly, even in those with very high risk with LDL-C > 190 mg/dl, an increase of one or two quintiles of *e*LDL-TG conferred an 18% and 41% increase risk, respectively (HR of 1.18 [95% CI, 1.03–1.34] and 1.41 [95% CI, 1.24–1.60]).

### 3.3 Discordance analysis of *e*LDL-TG and LDL-C

**Figure 3:**
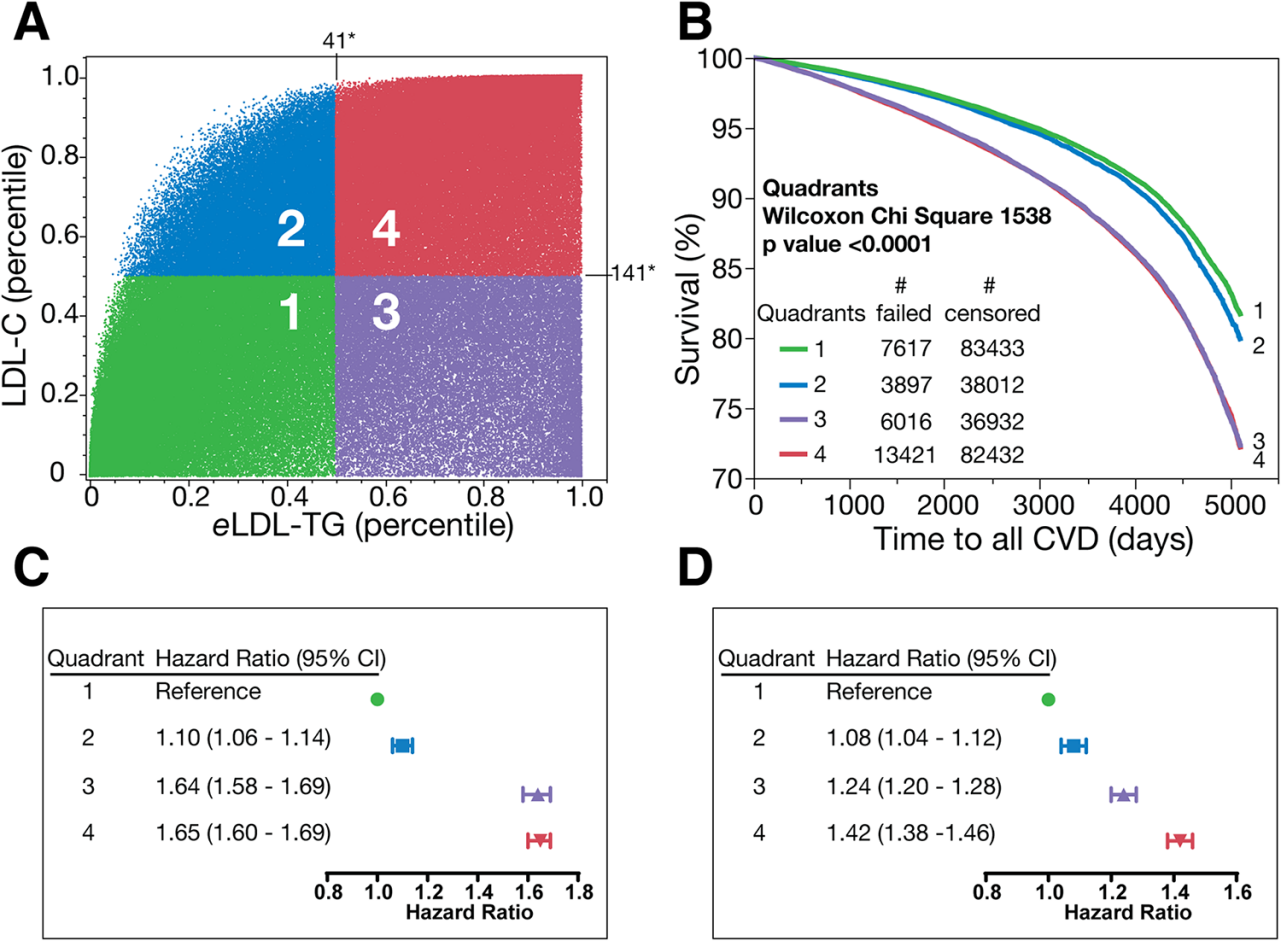
Discordance analysis and survival curves in the UKB dataset. Discordance analysis in UKB dataset for people without any lipid medications or missing follow-up data (*N* = 271,760). The percentile of LDL-C is plotted against the percentile of *e*LDL-TG and divided into quadrants (1-green, 2-blue, 3-purple, 4-red **(Panel A)** and Kaplan-Meier survival curves between these four quadrants are plotted in **Panel B**. **Panel C** shows unadjusted model for the ASCVD incidents per quadrants. **Panel D** shows model for the ASCVD incidents adjusted for nonlipid variables: age, sex, race, SPB, antihypertensive medication, diabetes, and smoking. * **(Panel A)** is concentration in mg/dl of the 50th percentile.

A simple discordance analysis was performed in the UKB cohort by plotting *e*LDL-TG against LDL-C and grouping individuals into one of 4 quadrants, which were created using cutpoints based on the 50th percentile of LDL-C (141 mg/dl) and *e*LDL-TG (41 mg/dl) (Figure 3A). One of the discordant quadrants (Quadrant 3: purple) with low LDL-C and high *e*LDL-TG (Figure 3A) and one of the concordant groups (Quadrant 4: red) with both high LDL-C and high *e*LDL-TG (Figure 3A) had the worst ASCVD outcomes and were nearly identical in their surivival curves (Figure 3B). Notably, Quadrant 2 (blue) with high LDL-C but low *e*LDL-TG had only slightly more ASCVD events than Quadrant 1 (green) with both low LDL-c and low *e*LDL-TG. This finding was confirmed when time to ASCVD event was considered and the HR was calculated by Cox proportional hazards analysis (Figure 3C). Quadrant 2 (high LDL-C/low *e*LDL-TG) had only a slightly higher unadjusted HR than Quadrant 1 (low LDL-C/low *e*LDL-TG). In contrast both Quadrants 3 (purple) and 4 (red), both of which had high *e*LDL-TG levels, had much higher HR than Quadrant 1 (green). After adjustment for non-lipid risk factors used in the 10-year pooled cohort equations (PCE) risk score (23), Quadrants 3 and 4 still had higher HR than Quadrant 2 (blue) consistent with *e*LDL-TG being a more predictive risk marker than LDL-C.

We next compared the independent associations of *e*LDL-TG with age and sex and other lipid and ASCVD risk markers for each quadrant in UKB cohort (Table 1). Quadrant 1 (green) had the lowest mean age, whereas Quadrant 4 (red) had the higest mean age but these differences were relatively modest compared to some of the other variables examined. Females were much more commonly found in Quadrants 1 (green) and 2 (blue), whereas males were more predominant in the other two Quadrants 3 (purple) and 4 (red). Individuals in Quadrants 3 and 4 with the highest ASCVD event rates (Figure 3A,B) also had higher levels of TG, *sd*LDL-C, SBP, BMI, glucose, haemoglobin A1C and C-reactive protein, and lower HDL-C compared to the other two quadrants. Finally, Quadrants 3 (purple) and 4 (red) also had higher 10-year PCE risk scores than the other quadrants. Although the mean values for the parameters differed likely due to differences in the age distribution of the population, similar assocations were found when individuals in the NHANES cohort were examined by the same quadrants based on LDL-C and *e*LDL-TG (Supplementary Table 2).

**Table 1:** Comparison between lipid and other test values among the four quadrants in the UKB dataset.

Variables	Quadrant 1 (green)	Quadrant 2 (blue)	Quadrant 3 (purple)	Quadrant 4 (red)
Sample size (*N*)	122,511	55,339	54,661	121,833
% Male	36.3*	30.6*	65.6*	45.5*
Age (years)	53.7 (8.3) D	56.4 (7.7) B	55.5 (8.2) C	57.1 (7.6) A
HDL-C (mg/dl)	60.6 (14.7) B	66.5 (14.3) A	45.1 (10.5) D	54.5 (12.1) C
TC (mg/dl)	193 (24.5) D	240 (19.9) B	209 (25.3) C	265 (31.3) A
TG (mg/dl)	96.1 (30.9) C	90.4 (20.9) D	254 (109) A	186 (75.7) B
NonHDL-C (mg/dl)	132 (19.4) D	173 (11.8) B	164 (23) C	211 (27.5) A
apoB (mg/dl)	85.4 (12.3) D	109 (8.6) B	99.4 (14) C	129 (16.8) A
LDL-C (mg/dl)	115 (18.7) D	158 (12.6) B	119 (18.3) C	177 (25.2) A
*sd*LDL-C (mg/dl)	31.7 (6.6) D	39.3 (5.2) C	48.2 (7.2) A	59.4 (10.3) A
*e*LDLTG (mg/dl)	33.9 (4.7) D	38.5 (2.6) C	52.9 (10.3) A	51.8 (7.7) B
SBP (mmHg)	133 (18.4) D	136 (18.8) C	140 (17.5) B	141 (18.4) A
BMI (kg/m^2^)	25.8 (4.5) C	25.8 (4.1) C	29 (4.8) A	28 (4.4) B
CRP (mg/L)	2.4 (4.7) C	2.0 (3.8) D	3.1 (4.6) A	2.8 (3.9) B
glucose (mg/dl)	89 (15.8) C	89.1 (11.9) C	93.2 (24.8) A	90.7 (16.1) B
HbA1C (%)	5.3 (0.5) D	5.3 (0.4) C	5.5 (0.6) A	5.4 (0.5) B
PCE score	4.6 (5.4) D	5.6 (5.3) C	8.7 (7.4) A	8.3 (6.4) B

Mean (sd) ANOVA—levels not connected by same letter are significantly different (*p* values all <0.0001).

* ChiSquare *p* value <0.0001.

We also show in Table 1 that those with high *e*LDL-TG (Quadrants 3 and 4) had a higher prevalence of components of the metabolic syndrome and other cardiovascular risk factors, such as higher SBP, BMI, hemoglobin A1C (HbA1C), and C-reactive protein (CRP). The prevalence of metabolic syndrome was higher in those with high *e*LDL-TG (63% and 37% for Quadrant 3 and 4, respectively) when compared to those with low *e*LDL-TG (8% and 4% for Quadrant 1 and 2, respectively).

### 3.4 Evaluation of relationship between *e*LDL-TG and other risk markers

**Figure 4:**
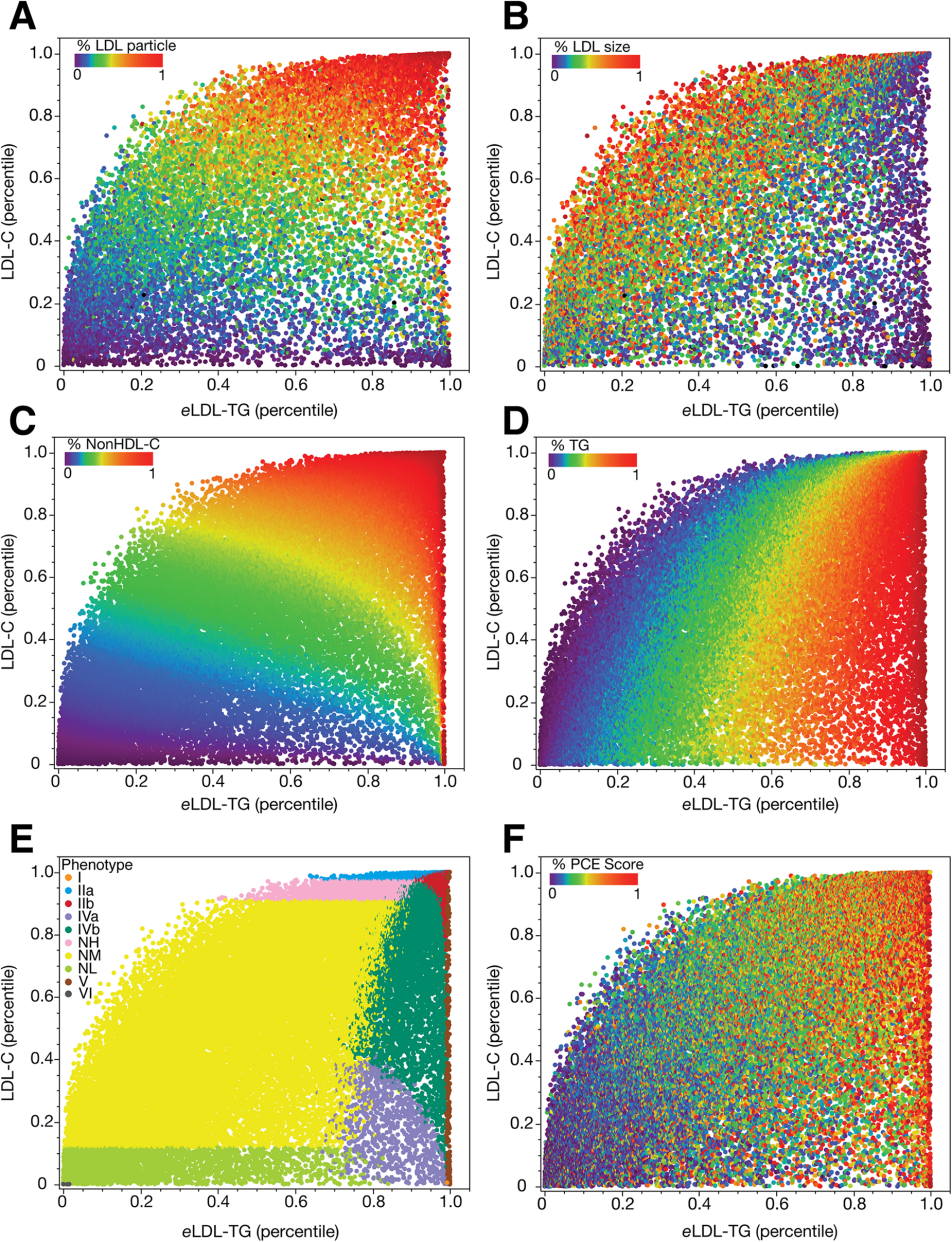
Percentile relationships between *e*LDL-TG and LDL-C in the NIH and NHANES datasets. Data is plotted as percentiles for *e*LDL-TG and LDL-C in an NIH dataset of NMR lipoprotein results (*N* = 13,793, **Panels A, B**). Results are colored by LDL particle number as a percentile **(Panel A)** and average LDL size as a percentile **(Panel B)**. Data is plotted as percentiles for *e*LDL-TG and S-LDL-C in NHANES **(Panels C–F)**. Results are colored by percentiles of nonHDL-C **(Panel C)**, percentiles of TG **(Panel D)**, lipoprotein phenotypes, according to the Sampson classification system **(Panel E)**, and the pooled cohort equations (PCE) calculated as if all patients were 55-year old White males and expressed in percentiles **(Panel F)**.

To further evaluate the relationship between *e*LDL-TG and ASCVD risk, we calculated *e*LDL-TG in NHANES and in a large general patient population cohort from the NIH, which were analyzed by NMR spectroscopy for lipoprotein subfractions (21) (Figure 4A,B). Total LDL particle number peaked in the top right corner of the plot and increased with both an increase in LDL-C and *e*LDL-TG (Figure 4A). In contrast, small in size LDL particles were more abundant on the right side of the plot and appeared to better overlap with *e*LDL-TG than LDL-C (Figure 4B). This is consistent with the known observation that most cholesterol is carried by large size LDL particles and that the number of large LDL particle decreases with increasing TG, wherease small LDL particles increase (15). When we grouped NHANES participants by their percentiles of nonHDL-C, we found that nonHDL-C increased with both increasing *e*LDL-TG and LDL-C but a subset of patients with the highest nonHDL-C levels showed a skewed distribution with more individuals having just high *e*LDL-TG (Figure 4C). An even more skewed distribution was observed for TG with the majority of hypertriglyceridemic individuals having high *e*LDL-TG levels regardless of their LDL-C levels (Figure 4D). When patients were grouped by their dyslipidemic phenotypes based on their lipid panel profile (24), more patients with high-risk phenotypes were found in parts of the plot with high *e*LDL-TG values (Figure 4E). Next, we calculated a 10-year risk score for the entire group by assuming each participant was a 55-year old White male (Figure 4F). Those with the highest risk score clustered better with their *e*LDL-TG values rather than LDL-C (Figure 4F).

### 3.5 Evaluation of *e*LDL-TG as a risk-enhancer test

**Figure 5:**
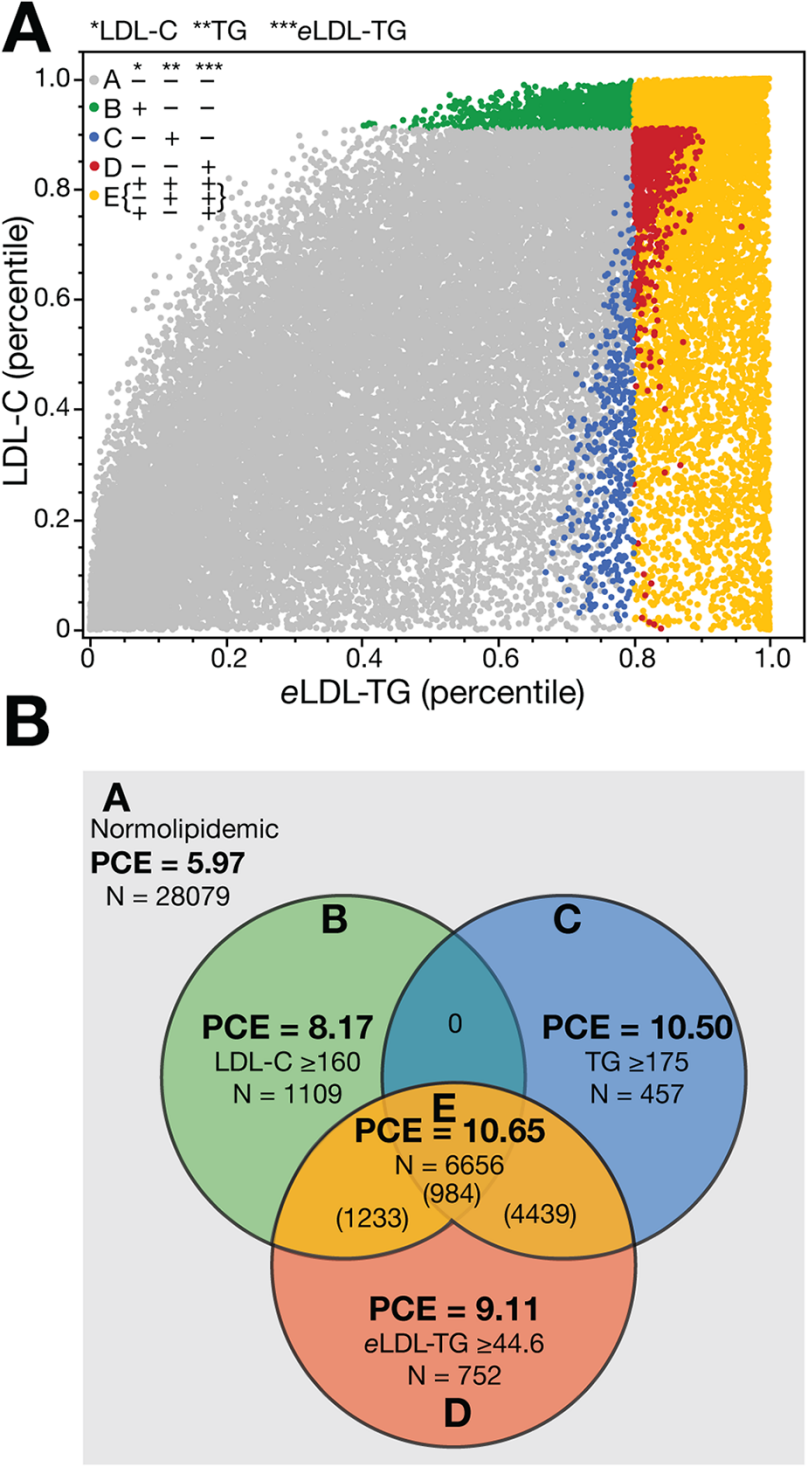
Evaluation of *e*LDL-TG as a risk-enhancer test in NHANES. The percentile of LDL-C is plotted against the percentile of *e*LDL-TG **(Panel A)** and shown in a Venn diagram **(Panel B)**. Points are colored by which risk-enhancer tests are present. Group A (light grey, *N* = 28,079) are normolipidemic patients below the cut-off for LDL-C (160 mg/dl; 4.14 mmol/L), TG (175 mg/dl; 1.97 mmol/L) and *e*LDL-TG (80th percentile, 44.6 mg/dl; 0.50 mmol/L). Group B (green, *N* = 1,109) are only positive for the LDL-C cut-off. Group C (blue, *N* = 457) are only positive for the TG cut-off. Group D (red, *N* = 752) are only positive for the *e*LDL-TG cut-off and represent a potentially unrecognized high-risk group. Group E (yellow, *N* = 6,656) is positive for both *e*LDL-TG and either LDL-C or TG, or all three tests.

We evaluated whether *e*LDL-TG could be used as a risk-enhancer test in the NHANES cohort (Figure 5). As before, the percentile of LDL-C was plotted against the percentile of *e*LDL-TG (Figure 5A), and we chose the 80th percentile (*e*LDL-TG ≥ 44.6 mg/dl; 0.50 mmol/L) as the cut-point. We then applied two commonly used risk-enhancer based on LDL-C and TG (2) for identifying higher-risk patients. Group A (grey) were defined as normolipidemic because they were negative for all the conventional lipid risk-enhancer tests and also had low *e*LDL-TG. All the other groups were positive for one or more of the lipid risk enhancement rules as described in the figure legend.

In Figure 5B, we tabulated the number in each group and found that the new *e*LDL-TG risk enhancer rule, when used in conjunction with the other two lipid risk enhancement rules, identifies approximately 50% more high-risk individuals. Furthermore, the mean 10-year risk score for those individuals only identified by the *e*LDL-TG cut-point is higher than what was observed for those patients only identified by the LDL-C > 160 mg/dl (4.14 mmol/L) risk enhancement rule (9.11 vs. 8.17; *p* = 0.054).

## 4 Discussion

We describe in the present report the first method for estimating LDL-TG with an equation, which like estimated LDL-C only uses variables from the standard lipid panel. Estimated LDL-TG by our new equation outperforms estimated LDL-C as a ASCVD risk marker in the primary prevention cohorts examined in our study. Currently, LDL-C is the main lipid marker used for initial ASCVD risk stratification. Hence, the findings from this and previous studies (15–18) on the stronger association of LDL-TG with ASCVD events than LDL-C could have a major impact in improving the diagnosis and treatment of cardiovascular disease.

The role of TG as an independent predictor of ASCVD has long been debated (25, 26), but more recent genetic type studies, such as genome-wide association and mendelian randomization studies, have shown that many genes that regulate TG levels are causally associated with ASCVD and to all-cause mortality (27, 28). Hypertriglyceridemia promotes the formation of small dense LDL particles, potentially increasing their atherogenicity (29). This occurs because of the transfer of TG from TRL onto LDL in exchange for cholesteryl esters by the Cholesterol Ester Transfer Protein (15, 30). When TG in the core of LDL undergoes lipolysis it leads to the production of smaller, denser LDL particles. A similar phenomena occurs with HDL particles, leading to an abnormal lipid and proteomic content and impaired vascular protective functions (31). The hydrolysis of LDL-TG by hepatic lipase (32) and lipoprotein lipase (33) in the vessal wall may also increase inflammation and promote atherogenesis.

Most previous studies that have explored the role of TG metabolism in cardiovascular disease have focused on TRL or their remnants derived from the partial lipolysis of apolipoprotein B-48 and apolipoprotein B100-containing lipoproteins, with less emphasis on exploring the role of the TG content of LDL. In a recent study by Balling et al. (17), a strong association between LDL-TG levels, as measured using the Denka direct assay, and increased incidents of ASCVD was found. This investigation was conducted in two large general population cohorts from the Copenhagen General Heart Study and revealed a strong association of direct LDL-TG with ASCVD, which included myocardial infarction, stroke, and peripheral artery disease. As we have observed, LDL-TG has been strongly linked to specific conditions that predispose to ASCVD, such as diabetes and obesity (34).

Several other lipid tests have been previously shown to be superior to LDL-C as an ASCVD biomarker (35). In the PREDIMED study (36), remnant cholesterol was a better ASCVD risk marker than LDL-C with hypertriglyceridemia. Similarly we found that *e*LDL-TG was more predictive than LDL-C but also superior to Rem-C and TGs. This may be because when Rem-C is estimated using LDL-C calculated by the Friedewald equation, it is mathematically the equivalent to ranking risk just based on the TG level (37). ApoB, the main protein component of LDL and TRL, is another marker that has been found in numerous studies to better predict ASCVD risk, particularly when discordant with LDL-C (38). This is likely because apoB, which provides a metric of the total particle number of atherogenic lipoproteins, does not decrease with hypertriglyceridemia unlike LDL-C (17). Although its use is well justified based on a cost-base analysis (39), it is still not widely utilized because it is not recommended at this time by most guidelines for initial risk evaluation (2). This prompted us to consider whether another metric, such as LDL-TG could serve as an alternative biomarker, particularly if it could be calculated from the same lipid parameters in the standard lipid panel, which are already used to calculate LDL-C. Although the correlation between *e*LDL-TG and directly measured LDL-TG was only modest, we show in two large cohorts that it was more strongly associated with ASCVD than estimated LDL-C. Unexpectedely, we also observed in the UKB cohort that *e*LDL-TG was superior to measured apoB or estimated *sd*LDL-C as a risk marker for ASCVD. We also show that *e*LDL-TG appears to increase ASCVD risk even above and beyond LDL-C > 190 mg/dl. In addition, the use of *e*LDL-TG as a risk-enhancer tests uniquely identifies high-risk patients who were not detected by other conventional lipid risk-enhancer tests. These findings suggest that *e*LDL-TG could possibly be calculated along with LDL-C and both could possibly be used for initial risk stratification and or *e*LDL-TG could be just utilized as a risk enhancer test. Based on our analysis, much of the association of *e*LDL-TG with ASCVD events may be due to its link with other high risk conditions, so in a fully adjusted, such as for the PCE that are used to predict 10-year risk, it is less likely to be useful.

It is important to note that our study has several limitations. In our investigation of the association of *e*LDL-TG with ASCVD events, we did not directly measure LDL-TG. Given the multiple factors that can potentially modulate LDL-TG levels, it is likely that directly measured LDL-TG would be superior to the *e*LDL-TG as an ASCVD biomarker but this will have to await future investigation. Nevertheless, until a direct LDL-TG assay is approved by the FDA and is available for routine testing, estimation of LDL-TG by our calculation method could be useful in the interim. Another limitation of our study is that we only examined the association of *e*LDL-TG with ASCVD in two cohorts. Before *e*LDL-TG can be recommended for routine use, it needs to be evaluated in many additional cohorts, and its potential clinical utility must be carefully examined. Additional studies will also be needed to clarify if *e*LDL-TG is causally related to ASCVD and if so the mechanism for how it promotes atherosclerosis. Such studies could reveal whether lowering LDL-TG could be a potential new drug treatment strategy for the prevention of ASCVD. The ability, however, to calculate LDL-TG by our method could facilitate future genome-wide association studies and Mendelian Randomization type studies to address this question. Eventually the effect of lipid-lowering therapy in reducing LDL-TG and its subsequent impact in cardiovascular outcomes should be directly examined.

In summary, we present an equation for estimating LDL-TG that does not require any additional laboratory testing. It is based on the results from the standard lipid panel and hence could be easily implemented by clinical laboratories for improving ASCVD risk prediction without increasing laboratory testing costs. More research and clinical studies, however, are needed in other populations to confirm the generalizability of our findings and to better understand its possible clinical utility.

## Data Availability

Researchers used five different datasets for this study. Two of them, NHANES and ARIC, are publicly available at https://wwwn.cdc.gov/nchs/nhanes/ and https://biolincc.nhlbi.nih.gov/studies/aric/, respectively. The other datasets, from the Mayo Clinic, NIH and UK Biobank, are accessible only to approved researchers through these institutions. Software for estimating *e*LDL-TG by the newly developed equation can be freely downloaded at the Figshare website (website: https://doi.org/10.6084/m9.figshare.23679375).

## Supplementary material

Supplementary Figure 1 Relationship between LDL-TG measured by the BQ reference method and apoB. Results for BQLDL-TG = 24,590 are colored by TG.

Supplementary Figure 2 Residual error plots comparing LDL-TG measured by the BQ reference method and estimated LDL-TG. Difference between LDL-TG measured by the BQ reference method (*BQ*LDL-TG) and the estimated LDL-TG (*e*LDL-TG) is plotted against NonHDL-C **(Panel A)**, TG **(Panel B)**, HDL-C **(Panel C)** and apoB **(Panel D)**. Results are colored by TG **(Panels A, C, and D)** and nonHDL-C **(B)**.

Supplementary Figure 3 Development of the equation for estimated LDL-TG (*e*LDL-TG) in SI units. Equation parameters are from the training dataset (*N* = 20,191) and graphed results are from the validation dataset (*N* = 20,011). Results are colored by TG.

Supplementary Figure 4 Kaplan-Meier survival curves and lipid test evaluation for ASCVD events in the ARIC dataset. Kaplan-Meier survival curves in ARIC for people without any lipid medications (*N* = 14,195) for all ASCVD events were calculated for LDL-C quintiles **(Panel A)** and *e*LDL-TG quintiles **(Panel B)**. Other lipid tests were divided into quintiles for survival curves and the Wilcoxon Chi-Square is ranked **(Panel C)**. The same set of lipid tests was evaluated with logistic regression for ASCVD and the AUC is ranked **(Panel D)**.

Supplementary Figure 5 Relationship between LDL-C and *e*LDL-TG. Results are colored by *e*LDL-TG quintiles.

Supplementary Table 1 Lipid values for the various datasets.

Supplementary Table 2 Comparison between lipid and other test values among the four quadrants in NHANES.
