# Supplementary figures and images for: An equation for estimating low-density lipoprotein-triglyceride content and its use for cardiovascular disease risk stratification
Source: Front Cardiovasc Med. 2024 Oct 25;11:1452869. doi: 10.3389/fcvm.2024.1452869 (PMC11543484; doi:10.3389/fcvm.2024.1452869)

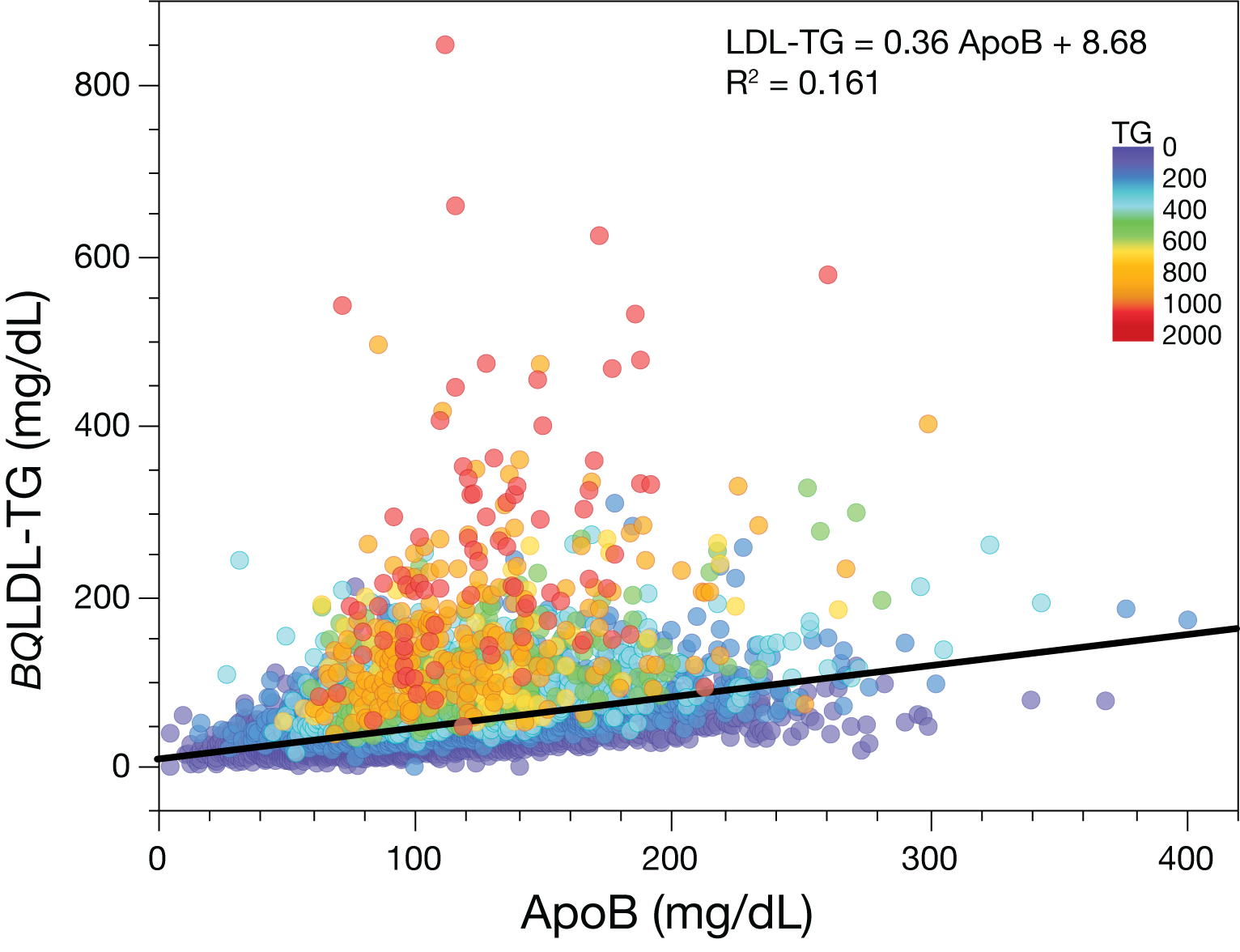

Supplement: Supplementary Figure 1 — Relationship between LDL-TG measured by the BQ reference method and apoB. Results for BQLDL-TG = 24,590 are colored by TG. [file Image1.tif]

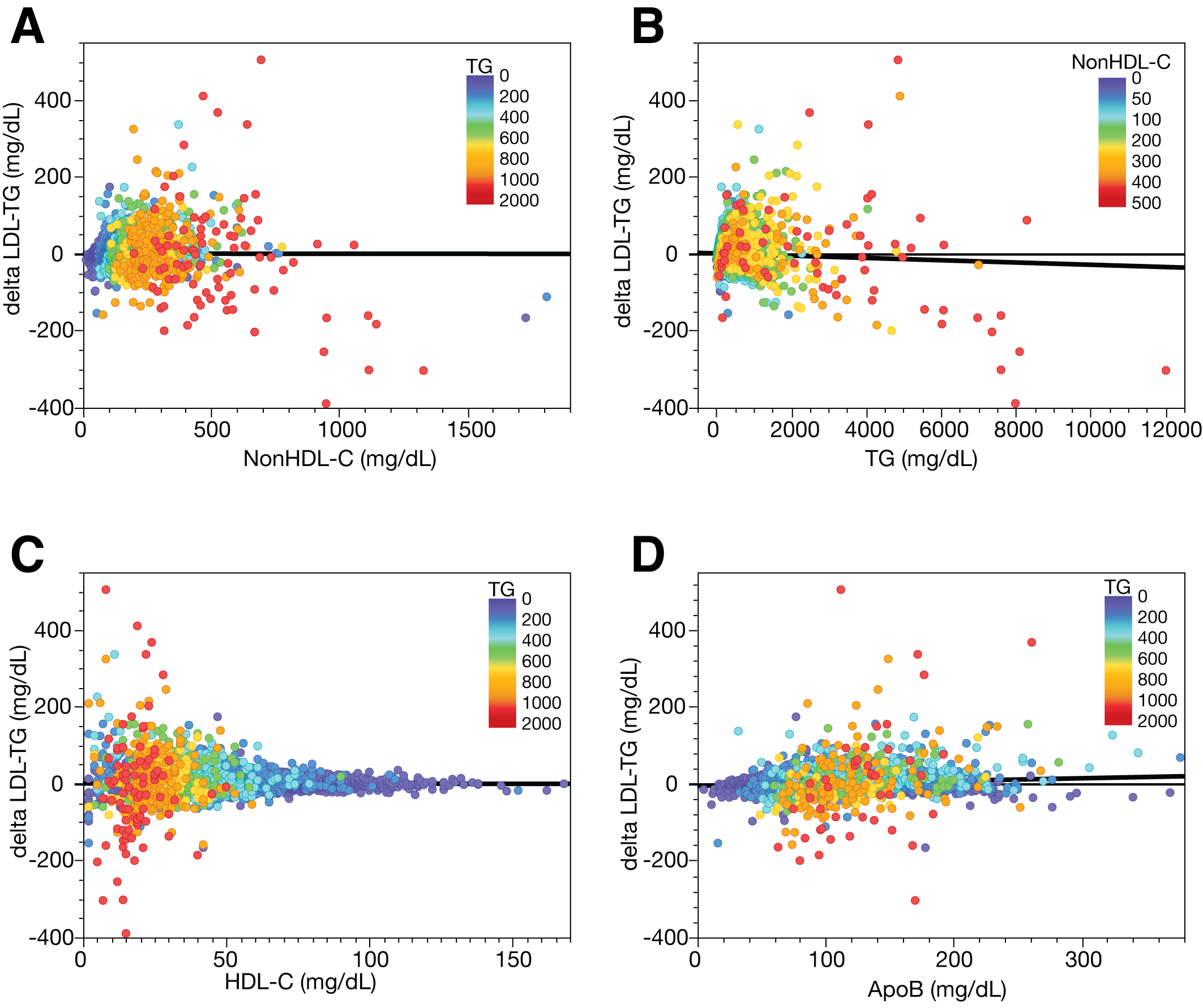

Supplement: Supplementary Figure 2 — Residual error plots comparing LDL-TG measured by the BQ reference method and estimated LDL-TG. Difference between LDL-TG measured by the BQ reference method (BQLDL-TG) and the estimated LDL-TG (eLDL-TG) is plotted against NonHDL-C (Panel A), TG (Panel B), HDL-C (Panel C) and apoB (Panel D). Results are colored by TG (Panels A, C, and D) and nonHDL-C (B). [file Image2.tif]

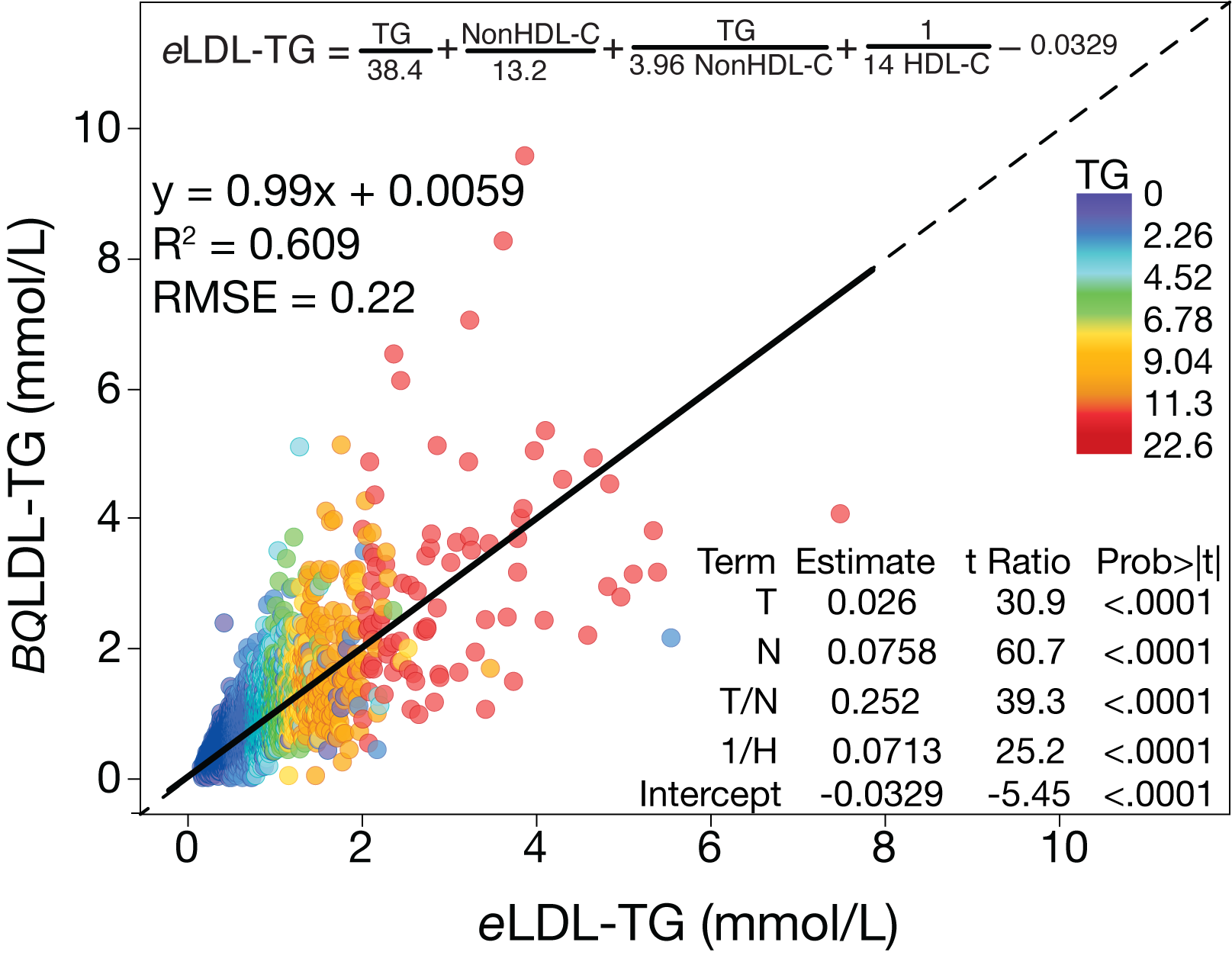

Supplement: Supplementary Figure 3 — Development of the equation for estimated LDL-TG (eLDL-TG) in SI units. Equation parameters are from the training dataset (N = 20,191) and graphed results are from the validation dataset (N = 20,011). Results are colored by TG. [file Image3.tif]

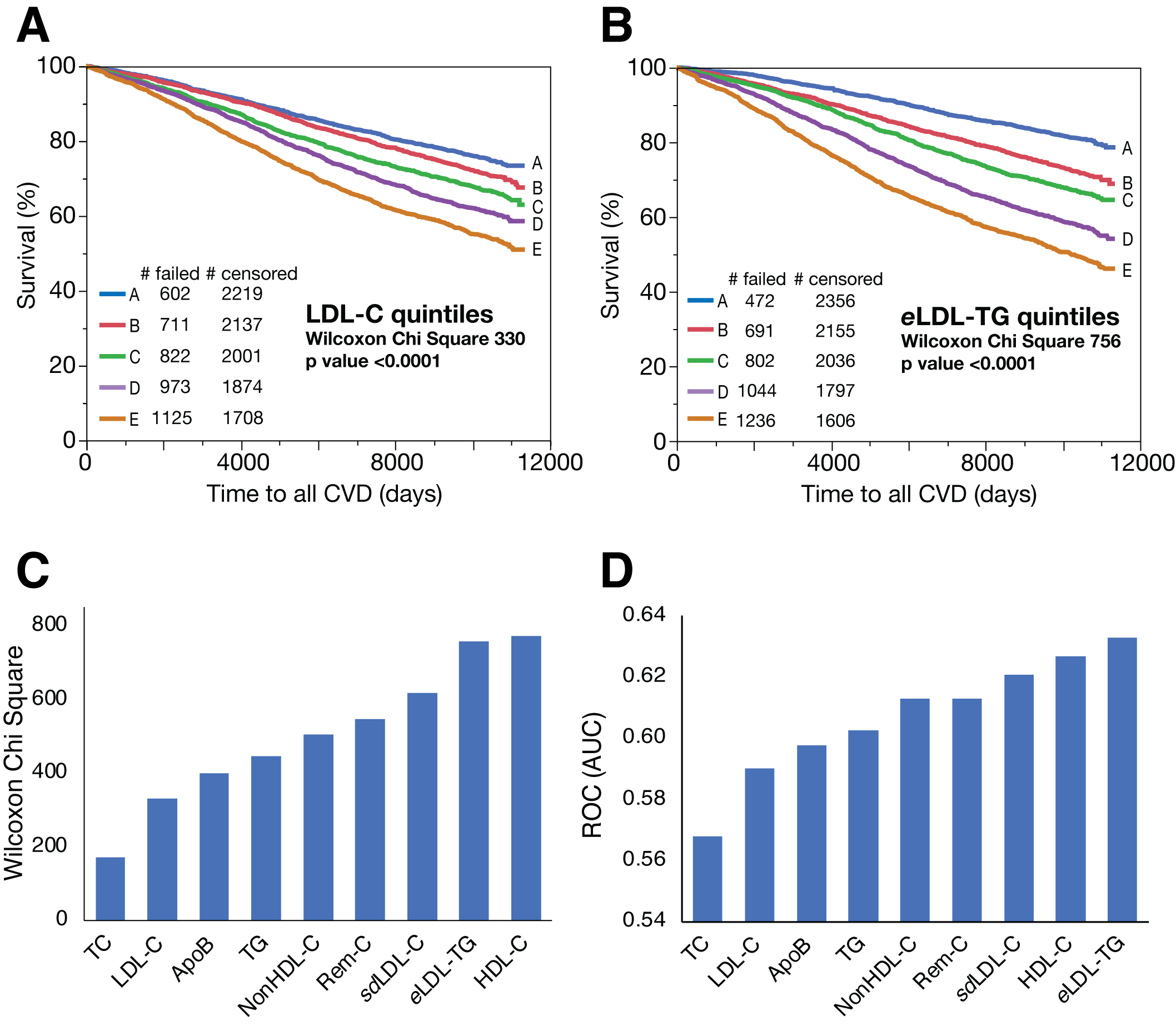

Supplement: Supplementary Figure 4 — Kaplan-Meier survival curves and lipid test evaluation for ASCVD events in the ARIC dataset. Kaplan-Meier survival curves in ARIC for people without any lipid medications (N = 14,195) for all ASCVD events were calculated for LDL-C quintiles (Panel A) and eLDL-TG quintiles (Panel B). Other lipid tests were divided into quintiles for survival curves and the Wilcoxon Chi-Square is ranked (Panel C). The same set of lipid tests was evaluated with logistic regression for ASCVD and the AUC is ranked (Panel D). [file Image4.tif]

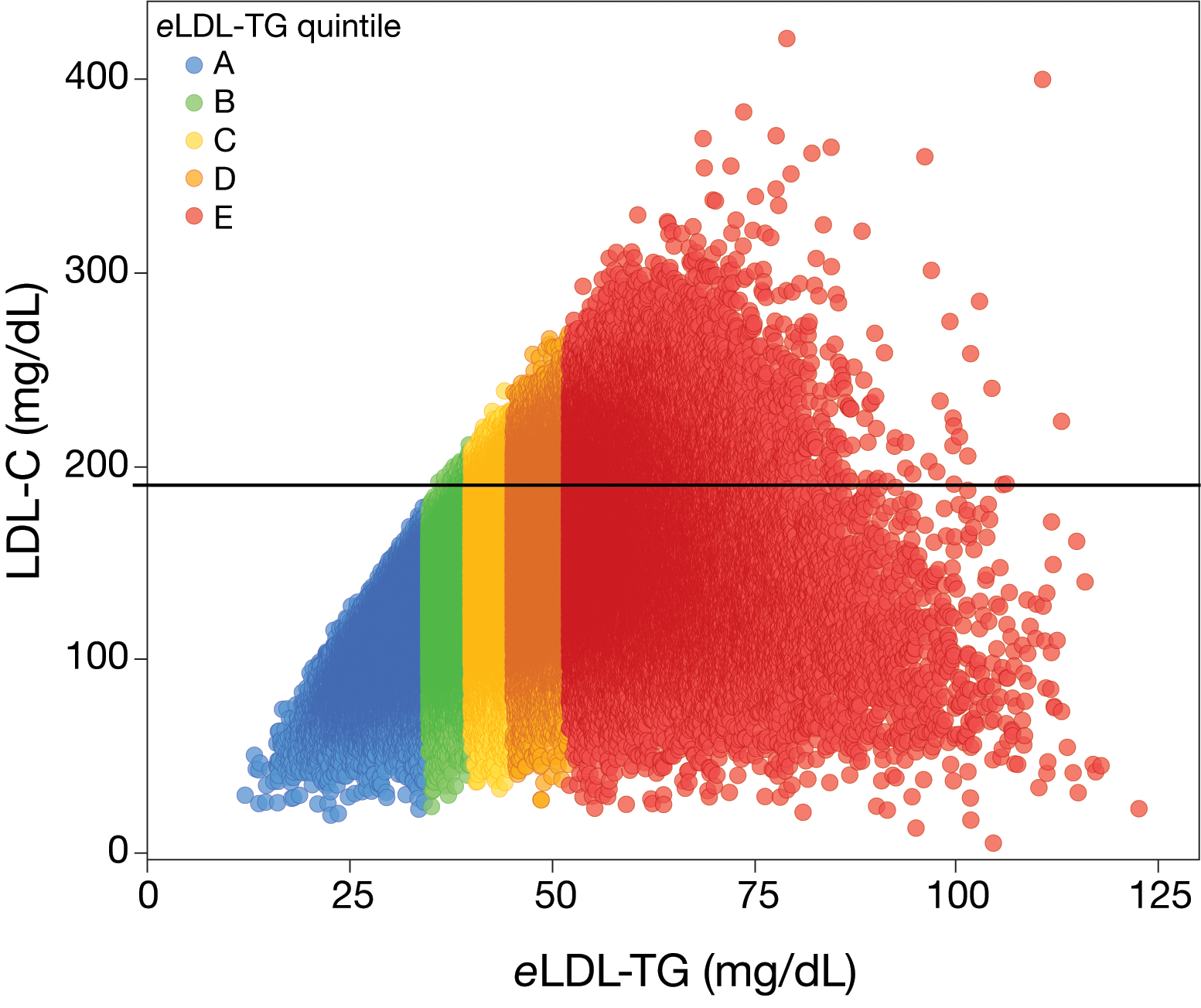

Supplement: Supplementary Figure 5 — Relationship between LDL-C and eLDL-TG. Results are colored by eLDL-TG quintiles. [file Image5.tif]
