# Supplementary material for: An equation for estimating low-density lipoprotein-triglyceride content and its use for cardiovascular disease risk stratification
Source: Front Cardiovasc Med. 2024 Oct 25;11:1452869. doi: 10.3389/fcvm.2024.1452869 (PMC11543484; doi:10.3389/fcvm.2024.1452869)
Supplement: Supplementary Table 1 — Lipid values for the various datasets. [file Table1.pdf]

Supplementary Table 1. Lipid values for the various datasets.

Variables	MML training	MML validation	NHANES	NIH	ARIC	UKB
Sample size (N)	20,191	20,011	37,053	13,788	14,195	271,760
Age (years)	54.6 (14.9)	54.5 (15.1)	40.9 (22.2)	47.8 (17.1)	54.2 (5.8)	56.3 (8)
HDL-C (mg/dL)	46 (15.1)	46.1 (15.6)	53.5 (15.3)	54.9 (19.6)	51.6 (17)	56.9 (14.7)
TC (mg/dL)	199 (66.6)	198 (64.9)	185 (42.5)	177 (47.9)	214 (41.6)	228 (41.1)
TG (mg/dL)	197 (319.6)	196 (304.4)	118 (100)	124 (116)	131 (87.7)	153 (89.1)
NonHDL-C (mg/dL)	153 (66.2)	152 (64.5)	132 (42.1)	122 (45.5)	163 (43.8)	171 (39.6)
apoB (mg/dL)	99.9 (31.8)	99.2 (32.3)	88.9 (26.2)	90.5 (30.4)	93.3 (28.8)	107 (23.1)
LDL-C (mg/dL)	118 (47.8)	117 (45.7)	110 (36.1)	99.7 (40.3)	139 (39.2)	144 (34.5)
% males	51.6	51.4	49.0	44.9	45.7	42.9
