# Supplementary material for: An equation for estimating low-density lipoprotein-triglyceride content and its use for cardiovascular disease risk stratification
Source: Front Cardiovasc Med. 2024 Oct 25;11:1452869. doi: 10.3389/fcvm.2024.1452869 (PMC11543484; doi:10.3389/fcvm.2024.1452869)
Supplement: Supplementary Table 2 — Comparison between lipid and other test values among the four quadrants in NHANES. [file Table2.pdf]

Supplementary Table 2. Comparison between lipid and other test values among the four quadrants in NHANES.

Variable	Quadrant 1 (green)	Quadrant 2 (blue)	Quadrant 3 (purple)	Quadrant 4 (red)
Sample size (N)	4309	2463	2743	8962
% Male	49.1*	40.1*	57.0*	49.3*
Age (years)	61.9 (13.2) A	58.6 (12.2) C	62.5 (12.3) A	59.2 (12.1) B
HDL-C (mg/dL)	61 (18.2) B	66.5 (16.7) A	44.1 (12.3) D	50.6 (13.4) C
TC (mg/dL)	157 (25.1) D	202 (21.6) B	167 (30.7) C	226 (34.8) A
TG (mg/dL)	74.5 (23.7) C	68.5 (17.1) D	221 (200) A	159 (84.9) B
NonHDL-C (mg/dL)	96.3 (17.4) D	135 (11.2) B	123 (30.6) C	175 (32.5) A
apoB (mg/dL)	69.3 (12.4) D	91.2 (9.9) B	83.4 (16.7) C	114 (20) A
LDL-C (mg/dL)	82 (17.2) D	123 (12.1) B	85.8 (16.8) C	146 (28.6) A
*sd*LDL-C (mg/dL)	22.3 (4.9) D	27.6 (4.4) C	34.4 (6.7) A	46.9 (11.4) A
*e*LDLTG (mg/dL)	27.8 (4.2) D	31.2 (2.3) C	46.7 (17.3) A	45.5 (9.5) B
SBP (mmHg)	128 (20.9) B	127 (20.6) C	130 (19.3) A	130 (20.4) A
BMI (kg/m^2)	28.4 (7) C	27.7 (6.5) D	31.2 (6.6) A	29.7 (6.3) B
CRP (mg/L)	4.7 (10.4) BC	4.2 (9.5) C	5.4 (10.2) A	4.9 (8.7) B
glucose (mg/dL)	112 (33.9) C	104 (24.8) D	130 (51.1) A	115 (41.6) B
PCE	13.4 (13.3) B	9.2 (10.3) D	16.8 (14) A	12.3 (11.6) C

With patients aged 40 and up only (N=18,477) * ChiSquare p value <0.0001
mean (sd) ANOVA – levels not connected by same letter are significantly different (p values all <0.0001)
